# The role of artificial intelligence and machine learning in predicting and combating antimicrobial resistance

**DOI:** 10.1016/j.csbj.2025.01.006

**Published:** 2025-01-18

**Authors:** Hazrat Bilal, Muhammad Nadeem Khan, Sabir Khan, Muhammad Shafiq, Wenjie Fang, Rahat Ullah Khan, Mujeeb Ur Rahman, Xiaohui Li, Qiao-Li Lv, Bin Xu

**Affiliations:** a Jiangxi Key Laboratory of oncology (2024SSY06041), JXHC Key Laboratory of Tumour Metastasis, NHC Key Laboratory of Personalized Diagnosis and Treatment of Nasopharyngeal Carcinoma, Jiangxi Cancer Hospital & Institute, The Second Affiliated Hospital of Nanchang Medical College, Nanchang, Jiangxi 330029, PR China; b Department of Cell Biology and Genetics, Shantou University Medical College, Shantou 515041, China; c Department of Dermatology, The Second Affiliated Hospital of Shantou University Medical College, Shantou 515041, China; d Research Institute of Clinical Pharmacy, Department of Pharmacology, Shantou University Medical College, Shantou 515041, China; e Department of Dermatology, Changzheng Hospital, Second Military Medical University, Shanghai 200003, China; f College of Life Sciences, University of Chinese Academy of Sciences, Beijing 101408, China; g Biofuels Institute, School of the Environment and Safety Engineering, Jiangsu University, Zhenjiang 212013, China; h CAS Key Laboratory of Pathogen Microbiology and Immunology, Institute of Microbiology, Center for Influenza Research and Early-warning (CASCIRE), CAS-TWAS Center of Excellence for Emerging Infectious Diseases (CEEID), Chinese Academy of Sciences, Beijing 100101, China

**Keywords:** Antimicrobial Resistance, Artificial Intelligence, Machine Learning, Drug Discovery, AMR surveillance

## Abstract

Antimicrobial resistance (AMR) is a major threat to global public health. The current review synthesizes to address the possible role of Artificial Intelligence and Machine Learning (AI/ML) in mitigating AMR. Supervised learning, unsupervised learning, deep learning, reinforcement learning, and natural language processing are some of the main tools used in this domain. AI/ML models can use various data sources, such as clinical information, genomic sequences, microbiome insights, and epidemiological data for predicting AMR outbreaks. Although AI/ML are relatively new fields, numerous case studies offer substantial evidence of their successful application in predicting AMR outbreaks with greater accuracy. These models can provide insights into the discovery of novel antimicrobials, the repurposing of existing drugs, and combination therapy through the analysis of their molecular structures. In addition, AI-based clinical decision support systems in real-time guide healthcare professionals to improve prescribing of antibiotics. The review also outlines how can AI improve AMR surveillance, analyze resistance trends, and enable early outbreak identification. Challenges, such as ethical considerations, data privacy, and model biases exist, however, the continuous development of novel methodologies enables AI/ML to play a significant role in combating AMR.

## Graphical Abstract

**Figure:**
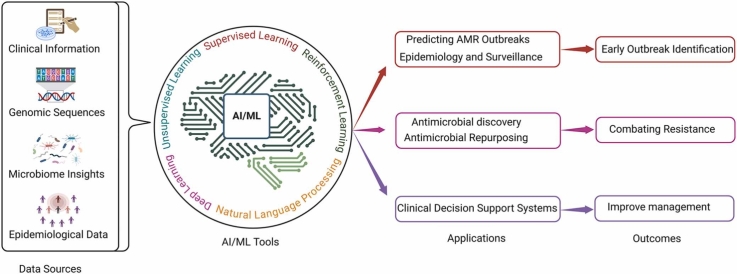

## 1 Introduction

Antimicrobial resistance (AMR) is recognized as a global public health crisis, driven by both epidemiological and economic factors, prompting the World Health Organization (WHO) to develop an action plan to tackle the issue [1]. Antibiotics are among the most frequently prescribed medications in both hospital and community settings. However, numerous prescriptions are either unnecessary or incorrect for instance, broad-spectrum antibiotics are often prescribed at inappropriate dosages or utilized for conditions that require targeted therapy [2]. This overuse or misuse of antibiotics resulted in the emergence of multi-drug resistant (MDR) pathogens. The rise of MDR bacterial infections significantly contributes to higher patient mortality, extended hospitalizations, and increased healthcare expenditures [3].

Over the past 15 years, considerable shortcomings have emerged in the development and availability of new antibiotics [4]. Antimicrobial stewardship, which involves the implementation of management strategies to control this rapidly growing concern is essential [4]. In previous years, a significant amount of clinical and laboratory data was either overlooked or not collected. Several reasons lead to this limitation, such as the complexity involved and the bulky nature of the data, lack of awareness, and absence of standardized protocol. However, the essay access to and availability of electronic health records (EHR) containing patient microbiological data now enable personalized antibiotic management [5].

Machine learning (ML) employs advanced methods to provide evidence-based decision-making and predictive tools by utilization of clinical and laboratory data [5]. Similarly, artificial intelligence (AI) enhances data analysis by rapidly processing information and providing rational decisions [6]. A well-designed AI algorithm can overcome human-associated limitations including neglecting instructions, weariness, and peer influence on hierarchical cultural norms. Based on their potential, AI and ML could significantly improve the research efficiency of complex health challenges like AMR [6]. This review comprehensively overviews various aspects of AI/ML in mitigating AMR including data analysis, predictive modeling, the discovery and design of new antimicrobials, surveillance, and clinical decision support systems. Additionally, we discussed the challenges, research gaps, and future directions in this emerging field.

## 2 AI/ML basics

AI is a branch of computer science focused on developing machines based on human intelligence, such as understanding learning, language processing, identifying patterns, resolving challenges, and reaching conclusions [7]. AI contains various branches that focus on specific functions, such as computer vision, expert systems, robotics, natural language processing, neural networks, fuzzy logic, and machine learning [8]. ML utilizes algorithms to perform tasks and enhances its performance by learning from input data [9]. The learning process of AI/ML is not limited by training data or time, enabling it to continuously improve its performance over time. Their ability to professionally process large datasets makes them ideal for addressing global AMR challenges [10]. AI/ML models can rationally analyze complex EHR data and predict patients who are likely to have AMR and those who may be at risk [11]. They can assist in drug designing, drug repurposing, and synergistic therapies by screening extensive libraries of chemical compounds against AMR pathogens [12]. They can identify the dissemination of AMR genes, identify risks, provide important tools to monitor public health and propose new diagnostic and therapeutic approaches [11].

The frequently used AI/ML techniques include supervised learning algorithms, unsupervised learning algorithms, reinforcement learning (RL), and deep learning (DL) architectures. Supervised learning algorithms use label datasets like patient records and microbial genomics, to make predictions and decisions. For example, Supervised machine learning has identified genetic traits associated with antibiotic sensitivity in *Escherichia coli* across various sequence types (STs). These genetic markers help elucidate how STs evolve and spread within populations that are likely to facilitate dissemination [13]. Similarly, it was used to predict *Streptococcus pneumoniae* susceptibility to β-lactam antibiotics by correlating penicillin-binding protein (PBP) sequences with minimum inhibitory concentrations (MIC) values [14]. Unsupervised learning algorithms do not rely on previously labeled or categorized data, instead focusing on independently identifying clusters and patterns within datasets. This approach can uncover new insights and hidden connections within microbial communities and resistant strains [15]. For example, a recent study used unsupervised learning and identified the simultaneous presence of antibiotic resistance and metal resistance in *Salmonella enterica* [16].

Reinforcement learning utilizes trial-and-error feedback to determine the most effective drug combination or antimicrobial treatment strategies for resistant strains, even with limited knowledge about bacterial population behaviors [17]. For example, a recent study used this approach for the treatment of sepsis, providing optimal antibiotic combination and duration that align with clinical practices [18]. DL methods such as convolutional neural networks (CNNs) and recurrent neural networks (RNNs) are used to identify complex patterns in data. CNNs can identify antibiotic susceptibility patterns from genome analysis while RNNs are capable of predicting antimicrobial resistance from time-series data [19]. The selection of AI and ML methods depends on the type of data source and objectives of the analysis. In addition to these common techniques, various specialized methods and algorithms, along with their advantages and disadvantages, are detailed in Table 1.

**Table 1:** AI/ML techniques and algorithm used in combating AMR.

Algorithm/Technique	Application	Advantage	Disadvantage
1. Supervised Learning Algorithms			
Logistic Regression	Prediction	Simple to implement and interpret	Assumes linear relationships
Decision Trees	Classification	Easy to interpret and visualize	Prone to overfitting
Random Forests	Resistance Prediction	Reduces overfitting and improves accuracy	Less interpretable and computationally intensive
Support Vector Machines (SVM)	Classification	Effective in high-dimensional spaces	Requires careful tuning
Gradient Boosting Machines (GBM)	Risk Assessment	High predictive accuracy	Complex and can overfit
AdaBoost	Classification	Improves weak classifiers and reduces bias	Sensitive to noisy data and outliers
Neural Networks (Deep Learning)	Genomic Analysis	Captures complex patterns	Requires large datasets and computational resources
2. Unsupervised Learning Algorithms			
K-Means Clustering	Pattern Recognition	Simple and efficient for large datasets	Requires predefined number of clusters
Hierarchical Clustering	Grouping	Dendrograms provide clear visual representation	Computationally expensive for large datasets
Principal Component Analysis (PCA)	Dimensionality Reduction	Preserves variance	May lose important information
t-SNE	Visualization	Effective for high-dimensional data	Computationally intensive
Autoencoders	Anomaly Detection	Learns efficient representations	Requires careful tuning
3. Semi-Supervised Learning Algorithms			
Semi-Supervised SVM (S3VM)	Classification	Utilizes both labeled and unlabeled data	More complex to implement
Self-training Algorithms	Resistance Prediction	Improves performance with minimal labeled data	Risk of propagating errors
4. Reinforcement Learning			
Q-Learning	Treatment Optimization	Effective for dynamic environments	Requires a lot of data
Deep Q-Networks (DQN)	Strategy Development	Combines deep learning with reinforcement	Computationally intensive
5. Ensemble Methods			
Bagging	Robustness	Reduces variance and improves accuracy	Can be less interpretable
Boosting	Performance Improvement	Reduces bias	Sensitive to outliers and noise
Stacking	Combined Predictions	Leverages multiple models	More complex and computationally intensive
6. NLP Techniques			
Text Mining	Literature Review	Extracts valuable information	Struggles with unstructured data
Sentiment Analysis	Public Opinion Analysis	Understands public sentiment	May misinterpret context
Topic Modeling	Research Trends	Identifies underlying themes	Results can be subjective
Named Entity Recognition	Data Extraction	Automates extraction of relevant information	Requires accurate training data
7. Neural Network Architectures			
Convolutional Neural Networks (CNN)	Image Analysis	Excellent for spatial data	Requires significant computational power
Recurrent Neural Networks (RNN)	Time-Series Analysis	Effective for sequence data	Prone to vanishing gradients
Long Short-Term Memory Networks (LSTM)	Sequence Prediction	Captures long-term dependencies	More complex than standard RNNs
8. Anomaly Detection Techniques			
Isolation Forests	Outlier Detection	Effective for high-dimensional datasets	May not perform well on small datasets
One-Class SVM	Rare Event Detection	Good for detecting anomalies	Requires careful parameter tuning
Local Outlier Factor (LOF)	Anomaly Detection	Identifies anomalies based on local density	Computationally intensive for large datasets
9. Feature Selection Techniques			
Recursive Feature Elimination (RFE)	Feature Reduction	Identifies the most relevant features	Computationally expensive for large datasets
Lasso Regression	Variable Selection	Performs variable selection and regularization	May exclude relevant features
Mutual Information	Important Feature Identification	Captures non-linear relationships	Can be computationally intensive
10. Data Augmentation Techniques			
SMOTE	Imbalance Handling	Balances classes by generating synthetic examples	May introduce noise
Random Oversampling/Undersampling	Data Balance	Simple to implement	Can lead to overfitting or loss of information
11. Bayesian Approaches			
Bayesian Networks	Probabilistic Reasoning	Clear graphical representation of relationships	Computationally intensive
Naïve Bayes Classifier	Classification	Fast and works well with small datasets	Assumes feature independence
12. Model Evaluation Techniques			
Cross-Validation	Model Validation	Reliable estimate of model performance	Can be computationally expensive
ROC Curves and AUC	Performance Measurement	Insight into trade-off between sensitivity	May be misleading in imbalanced datasets
Confusion Matrices	Error Analysis	Detailed insight into classification errors	Limited to binary and multi-class classifications
13. Optimization Techniques			
Genetic Algorithms	Parameter Tuning	Effective for complex optimization problems	May require significant computational resources
Particle Swarm Optimization	Model Optimization	Simple to implement	Sensitive to parameter settings
14. Other Emerging Techniques			
Federated Learning	Collaborative Learning	Decentralized model training without sharing	Requires complex coordination
Transfer Learning	Model Adaptation	Leverages pre-trained models	Performance depends on similarity between tasks

## 3 Data sources for predicting AMR

AI/ML requires high-quality and reliable datasets from multiple sources to effectively address and predict AMR [11]. Surveillance networks, such as the European Antimicrobial Resistance Surveillance Network (EARS-Net) and the National Antimicrobial Resistance Monitoring System (NARMS) in the United States (US) actively monitor AMR by collaborating with clinical laboratories to collect data on antimicrobial susceptibility [20], [21]. Compressive initiatives like the Global Antimicrobial Resistance Surveillance System (GLASS) contribute to the global collection of AMR data [3]. Furthermore, large information centers like the Bacterial Isolate Genome Sequence Database (BIGSdb) and the Pathosystems Resource Integration Center (PATRIC) specialize in the whole genome sequencing (WGS) data of bacteria [22], [23]. Factors such as data size, robustness, and reproducibility must be assessed when collecting AMR data for processing using AI/ML algorithms [24]. This section discussed the main data sources and quality challenges associated with processing AI/ML models.

### 3.1 Clinical data

Clinical data refers to information related to patient medical records, typically available in the form of EHR. This data provides valuable insights into patient history, antimicrobial usage, and their effectiveness in relation to AMR strains. Such information is important for clinical decision support systems (CDSS) and helps healthcare professionals in prescribing appropriate treatment [25]. Supplementary information, such as antimicrobial susceptibility test (AST) results and genetic determinants, is essential alongside clinical data to improve AMR forecasting [26].

Researchers utilized ML models, incorporating algorithms such as Logistic Regression (LR), Support Vector Classifier (SVC), Random Forest (RF), eXtreme Gradient Boosting (XGBoost), K-Nearest Neighbor, (KNN), and Multilayer Perceptron (MLP), to identify intensive care unit (ICU) patients at risk of MDR pathogens based on EHR data. The Area under the receiver operating characteristic (AUROC) scores of 0.786 and 0.744 were obtained across two datasets collected within 24 hours of ICU admission [27]. Similarly, a study used ML algorithms, including LR, bootstrapping simulations, and gradient-boosting decision trees (GBDT), to analyze the correlation between urinary tract infection (UTI)-associated AMR and factors such as patient demography, empirical therapy, and urine culture results. An AMR predictive model incorporating the evaluated factors was developed, demonstrating the potential for recommending personalized treatment options. Using these algorithms during a one-year testing period reduced mismatched treatment rates to 5.1 %, representing a 42 % decline compared to treatments prescribed by physicians. This approach illustrates the potential for customizing antibiotic prescriptions to minimize treatment discrepancies in managing UTIs [28].

### 3.2 Epidemiological data

Epidemiological data encompasses information on disease outbreaks, the geographical spread of resistance, and the mechanisms of dissemination. Epidemiological databases offer insights into pathogen transmission within healthcare environments and communities. AI and ML models can improve the accuracy of predictions concerning the spread of resistance, leading to more effective interventions and optimized resource allocation [11].

A study analyzed data from five AMR surveillance projects conducted between 1997 and 2015. The objective was to model the progression of AMR across various combinations of countries, bacterial species, and antibiotics, to forecast the spread of AMR by examining temporal and geographical patterns. Out of 7440 triads analyzed, 14 % conformed to a sigmoid (logistic) model, revealing varying rates of resistance spread, including slow, intermediate, or rapid progression. Sigmoid models outperformed linear models in 84 % of instances, showing only a 6.5 % deviation from the observed data. The sequence-based approach is an innovative approach for predicting the spread of AMR, which can offer important information for healthcare policy-makers and pharmaceutical firms [29]. In another study, researchers used population-level health data across 259 countries and territories from 2000 to 2016 to forecast the spread of AMR. Researchers used ML techniques to predict global AMR trends using over 1500 metrics from the World Bank Health Nutrition and Population dataset. The findings revealed that up to 89 % of the observed variation in AMR could be attributed to general health and sanitation factors. They observed high AMR levels in Tanzania and Vietnam, while the Netherlands and Sweden exhibited low AMR levels [30]. Another study examined global AMR at a geopolitical territorial level, using data from 103 countries. This research utilized data from intercontinental medical statistics and quintiles (IQVIA) multinational integrated data analysis system (MIDAS), The World Bank DataBank, and Transparency International, covering the period 2008–2014, employing logistic regression and other predictive models for analysis. They compared socioeconomic characteristics, governance scores, and key health system variables for all included countries. This study found a significant association between reduced AMR and improved governance and infrastructure, although antibiotic consumption alone did not influence the rate of AMR [31].

Furthermore, AI may be used to develop early warning systems that predict the probability of outbreaks based on historical & risk factor-associated data [32], [33]. For example, Sundermann et al., in 2022 developed Enhanced Detection System for Healthcare-Associated Transmission (EDS-HAT) using ML models. This system utilized WGS data and patients’ health records to identify the transmission route of hospital-acquired infections and outbreaks [34]. AI-based image processing algorithms enable us to determine antibiotic susceptibility by measuring the zone of inhibition on culturing plates. This procedure is both cost-and time-saving and the final results can be directly submitted to WHO’s GLASS [35]. Similarly, HealthMap (www.healthmap.org, accessed on 25th December 2024), a real-time internet-based infectious disease surveillance system, utilizes nine languages to extract geographical data and identify unreported clusters of infectious diseases. These tools provide public health professionals with valuable information to support effective policy-making processes [36].

### 3.3 Genomic Data

Whole genome sequencing, metagenomics, and transcriptomics data are essential for understanding the diverse mechanisms underlying antibiotic resistance. The use of AI/ML techniques aids in identifying resistance genes and mutations/variations, enabling the study of AMR development and supporting personalized treatment recommendations. [37]. The knowledge derived from genomic data for predicting AMR is expanding with increased accessibility of this information. However, specialized expertise is required for its complex interpretation [38].

A study presents a framework for predicting drug resistance in *Mycobacterium tuberculosis* (MTB) through whole-genome mutations using a DL model developed with TensorFlow 2 application programming interface (API). The framework assessed essential elements of CNN, Denoising Autoencoders, and Wide & Deep models. A de novo learning strategy leveraging genome-wide mutations addressed the limitations of previous models, achieving robust performance with a sensitivity of 0.90 and specificity of 0.87. This model is accessible through tuberculosis drug resistance optimal prediction (TB-DROP) (https://github.com/nottwy/TB-DROP, accessed on 12th June 2024) [39]. Further research used advanced ML algorithms to predict the MICs of 13 antimicrobial agents against *Acinetobacter baumannii*. They utilized RF, SVM, and XGBoost with k-mer features extracted from WGS data. Among 339 isolates, the overall prediction rates of the models exceeded 90–95 %, except for levofloxacin, minocycline, and imipenem. The study found that feature selection pipelines achieved an optimal balance between training time and predictive performance. Specifically, extracting top-ranked 11-mers enabled predictions to be generated in about 10 minutes, with only a slight reduction in accuracy (96 %) when evaluated on an independent test set of approximately 120 newly sequenced isolates. It suggests that k-mer-based ML approaches are promising as robust and predictive tools for MIC prediction in clinical settings [40]. Similarly, an investigation involving 414 drug-resistant *Pseudomonas aeruginosa* isolates utilized ML classifiers, mainly SVM, to predict AMR using genomic and transcriptomic data. This study identified resistance biomarkers for four major antibiotics (tobramycin, ciprofloxacin, meropenem, and ceftazidime) by developing predictive models that incorporate gene presence/absence, sequence variation profiles, and expression status. Sensitivity rates and predictive values were observed to be in the high (0.8–0.9) to very high (> 0.9) ranges, with gene expression improving performance for all drugs except ciprofloxacin. These findings highlight the importance of molecular resistance profiling to enable early and accurate diagnosis in clinical microbiology [41].

### 3.4 Microbiome Data

Microbiomes consist of millions of microorganisms and play a crucial role in our health. Metagenomic and meta-transcriptomic data provide valuable insights into the taxonomic abundance and potential activities of microbes. AI/ML methods have the potential to elucidate the link between microbiome alteration and AMR development [42]. They can also predict the impact of interventions such as probiotics on AMR development within the microbiome [43]. Studying microbiome data can add significant value in predicting and combating AMR. Predicting AMR from microbiome data can guide preventive measures and targeted treatments for resistant strains [44].

Researchers used ML algorithms such as RF and GBDT to identify antibiotic-resistant genes (ARGs) in the gut microbiome of preterm infants. The analysis revealed that outer membrane proteins (OprC and OprD), associated with class D β-lactamases were crucial for bacterial survival under antibiotic exposure. The algorithms predict microbiome alterations and adaptability in response to cephalosporin and vancomycin [45]. A DL model was used to analyze ARGs within shotgun metagenomes and metagenome-assembled genomes (MAGs) obtained from the International Space Station (ISS). This approach enabled the identification of ARGs in both environmental samples and isolated bacterial strains. This model identified the dominance of ARGs in *Kalamiella piersonii*, a species associated with UTIs. Furthermore, computational analysis of 226 cultivable strains identified hundreds of ARGs, with notable prevalence in *Enterobacter bugandensis* and *Bacillus cereus*. The AMR predictions were validated experimentally through AST, which confirmed resistance to β-lactams antibiotics in these strains. This study demonstrated that a combined approach of computational and experimental validation can uncover hidden ARGs within complex environmental systems [46]. Another study used shotgun metagenomics with multivariate modeling to assess the impact of ciprofloxacin and cotrimoxazole on gut microbiota and resistomes in two cohorts of hematological patients. The antibiotics reduce microbiome diversity and have varying effects on resistomes. The cotrimoxazole-treated group has significantly elevated levels of sulfonamide ARGs and plasmids harboring ARGs compared to the ciprofloxacin-treated cohort. These findings underscore the predictive potential of ML algorithms, aiding in the implementation of targeted therapy [47].

### 3.5 Predictive Models for AMR

Predictive models using AI/ML algorithms provide quick information about resistant pathogens, aiding in the prompt mitigation of AMR. AI-based predictive models are essential for monitoring AMR patterns, as AI algorithms excel at uncovering hidden connections from data, which would take significantly longer for humans to analyze. Among the most important applications for enabling effective AI in AMR are time series analysis, phylogenetic studies, and network analysis. Predictive models can detect and alert atypical behaviors or clusters associated with drug resistance or overexpression of ARGs. Early detection facilitates the implementation of measures that help mitigate the risk of transmission and prevent further progression [11]. AI technology enables the analysis of archived AMR data to identify trends, seasonal variations, and potential outbreaks of resistance, thereby improving resource allocation in healthcare [48]. In addition, AI and ML enable more accurate construction of phylogenetic trees and facilitate surveillance of genetic lineages across pathogens, providing a deep understanding of resistance spread in hospitals and communities. These methodologies are capable of monitoring AMR transmission networks among patients, within wards, and across entire hospital systems. Additionally, they facilitate the identification of point-source outbreaks and healthcare-associated outbreak detection, aiding in timely intervention and containment strategies [49].

**Fig. 1:**
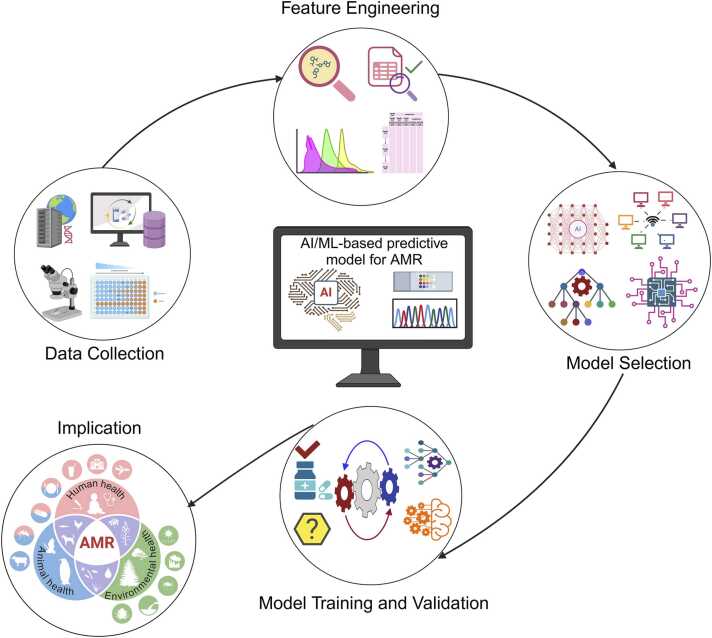
Steps Involved in Designing an Artificial Intelligence and Machine Learning-Based Predictive Model for Forecasting Antimicrobial Resistance.

Designing an AI/ML-based predictive model for AMR involves key steps, such as data collection, feature engineering, model selection, training, and validation before implementation (Fig. 1) [50]. An important aspect of model development is a careful selection of suitable data sources, which provide a solid framework for analysis. The data sources could include clinical, genomic, microbiomics, or epidemiological data, depending on the objective of the model [51]. To create an effective AI/ML-based model, high-quality data that is accurate, consistent, complete, and timely is required. Following quality standards and conducting regular audits are important for improving data integrity [52]. Features such as patient records, treatment outcomes, WGS, ARGs, single nucleotide polymorphism (SNP), outbreak information, and k-mers extraction and transformation are important for an AMR prediction model [53].

AMR prediction can be performed both phenotypically and at the molecular level, requiring different datasets and models for each approach. The dataset for phenotypic prediction primarily includes AST profiles, such as MIC values or zone of inhibition, along with patients’ demography like age and gender, underlying health condition, treatment outcomes, clinical sample types, and species information [54]. Molecular prediction requires genetic factors obtained from whole genome or metagenomic sequencing [55]. This data provides information related to specific mutations such as S639F mutation in the FKS1 gene in echinocandins resistant *C. auris* [56], or genes associated with resistance like *bla*_NDM_, and *bla*_KPC_ for carbapenemases production and *bla*_CTXM_, *bla*_TEM_, and *bla*_SHV_ for extended-spectrum β-lactamases (ESBL) production in Enterobacterales [57]. The absence or presence of these resistant determinates can predict the efficacy of treatment for infections. Moreover, environmental factors such as humidity and temperature can influence the spread of resistance and these data can also be incorporated into prediction models [58]. Various AI and ML methods (as mentioned in Table 1) can be applied to build predictive models. Data characteristics, such as size, distribution, and type can inform the suitability of the model. For example, simpler datasets may be better suited for LR, whereas more complex datasets may require a DL approach. Similarly, the predictive goal such as optimizing sensitivity or specificity, guide the selection of appropriate model [59]. Supervised learning algorithms, such as RF model identify patterns correlating specific pathogen traits with resistance profiles, including phenotypic AMR prediction [54]. For molecular based AMR prediction, the DL algorithms like CNNs are particularly effective. These algorithms can predict phenotypic outcomes by analyzing and identifying patterns associated with mutated or resistance genes [60].

The predictive performance of various ML models may vary depending on the choice of model selection, performance metrics used, characteristics of the dataset, and underlying assumptions [61]. A study utilized four different algorithms including LR, SVM, RF, and CNN to predict AMR using WGS data with various encoding methods. All models successfully predicted AMR on independent datasets; however, the RF demonstrated the best performance, followed by CNNs, LR, and SVM achieving the Area under curve (AUC) of up to 0.96 [62]. The selection of a specific model should be task-specific-adaptation based on their design and training methodology. For example, CNNs are well-suited for image-based diagnostics, while RNNs showed superior performance on sequential data [19]. Regarding the performance metrics, the AUC-ROC is a commonly used method; however, other metrics such as recall, F1 score, and precession also provide valuable insight, particularly for rare resistance phenotypes and specific types of AMR infection [63]. The AUC-ROC is well-suited for assessing binary classification with imbalance classes. However, in highly imbalanced datasets, it may not fully capture nuances. In such scenarios, the precession and recall might be more informative [64]. Precession is useful when the cost of false positives is higher, as it helps predict the true number of resistant cases. Recall focuses on identifying all relevant instances, ensuring accurate prediction of actual positive cases [65]. The F1 score is the harmonic mean of precession and recall and can maintain a balance between false positive and false negative cases [66]. To address complex scenarios like AMR, a holistic approach that incorporates all performance metrics is more effective in providing a comprehensive understanding. It is important to validate the robustness of predictive models for which cross-validation and performance metrics should be used to compare against alternative models [67].

For real-world applicability, integrating AI/ML-based models into existing systems is pivotal. Ensuring the compatibility of algorithm models with systems like EHR may require APIs or middleware solutions. Additionally, training healthcare staff is necessary for adapting workflow accordingly [68]. Besides these, ethical considerations are vital for the deployment of AI/ML-based models in healthcare settings. Data privacy and security of patient information must be protected and informed consent from patients should be obtained. Algorithmic bias must be addressed, and training data should include diverse datasets from various demographic regions to ensure consistency. Transparency among healthcare providers and patients, along with accountability between healthcare providers and stockholders, is essential for maintaining high standards [69].

### 3.6 Predictive Modeling for Priority Pathogens and Infections

The WHO announced priority bacterial (https://www.who.int/publications/i/item/9789240093461,) and fungal (https://www.who.int/publications/i/item/9789240060241, accessed on 25th December 2024) pathogen lists to strengthen global response to their infections and AMR. Predictive modeling for AMR must prioritize pathogens of global concern. Some key pathogens among the lists include methicillin-resistant *Staphylococcus aureus* (MRSA), carbapenemase-producing *Enterobacterales* (CPE), carbapenem-resistant *A. baumannii* (CRAB), Vancomycin-Resistant *Enterococcus faecium* (VRE), *P. aeruginosa*, and *C. auris* [70]. The data source for each type of resistant pathogen depends on the risk factors associated with their dissemination, while the model section depends on the type of data and predictive goals. For example, in the prediction of MRSA, data such as hospital stay duration, empirical therapy, and comorbidities are significant factors [71], [72]. The LR model can estimate the likelihood of MRSA infection based on empirical therapy, while RF enhances prediction accuracy by combining multiple decision trees to minimize overfitting [72], [73]. The data related to patient travel history and prior carbapenem usage can improve the predictive modeling of CPE [74]. SVM and Gradient Boosting Machines (GBM) models effectively identify risk factors associated with CPE [75]. Patient-specific data such as ICU stay and device use during the treatment can improve the predictive modeling of CRAB [76]. Neural networks can identify complex relationships between patient data and CRAB resistance, while XGBoost efficiently manages missing data and emphasizes key features [77]. For VRE, the information related to prior glycopeptide usage, immunosuppressive therapy, device-associated infection, and colonization pressure can enhance the accuracy of the prediction model [78]. RF and Decision trees can facilitate the interpretation related to patients’ history for VRE predictive models [79]. Similarly, for *C. auris* and other related yeast predictive models, data on local epidemiology, antifungal usage, and immunosuppressive therapy are important [80]. Bayesian logistic regression models have shown high sensitivity and specificity in predicting *C. auris* from genomic data [81]. Similarly, CNNs have demonstrated prominent results in identifying *Candida* species based on wet-mounted images [82].

Furthermore, for predicting infection types, specific information from EHR is required to improve the accuracy. For example, information such as broad-spectrum antimicrobial usage and invasive procedures are vital in cases of bloodstream infections (BSIs) [83], whereas catheterization history and recurrent infection patterns are important for UTIs [84]. DL and RF models analyze prior antimicrobial usage and clinical intervention to predict the likelihood of BSIs caused by resistant organisms [85]. SVM can classify patients based on UTI risk factors, while LR estimates the odds of resistance from past infections [86]. Similarly, for the surgical site infections (SSI) prediction model, data related to empirical therapy and surgery type is pivotal [87], while respiratory tract infection (RTI) models benefit from data on ICU admissions and mechanical ventilation [88]. Neural networks analyze complex interactions between prior therapeutical protocols and patient conditions to effectively predict RTI outcomes [89]. LR and KNN can predict SSI risk using surgical data and antibiotic strategies, helping to prevent infections after surgery [87].

### 3.7 Advantages and Disadvantages of AI/M-based Predictive Models

The main advantage of an ML-based predictive model is its ability to handle large and complex datasets, which classical statistics may find challenging. For example, a study predicts AMR in *Campylobacter* species by utilizing MALDTI-TOF MS protein mass spectra data, achieving a sensitivity of 92.3 and a precision of 81.2 % [90]. ML models can identify non-linear relationships, whereas classical models generally assume linear relationships. For instance, researchers used decision tree analysis to predict AMR in *E. coli,* influenced by environmental factors and antibiotic exposures [91]. Adaptability is a key feature of ML models, as they can be retrained with new data, allowing them to adapt to evolving patterns in AMR and improve the predictive accuracy over time with new data. RNNs can predict AMR trends using time-series data, such as microbial evaluation and treatment history [92]. The automation capability of ML models reduces the time required for real-time decision-making in antimicrobial surveillance. For example, the AutoMated tool for Antimicrobial Resistance Surveillance System (AMASS) quickly generates AMR reports from clinical laboratory data. This enables healthcare providers to respond quickly and effectively to emerging AMR outbreaks, thereby improving patient outcomes [93].

One of the main disadvantages of ML models is their Black Box nature, particularly concerning, as DL algorithms are often criticized for their lack of interpretability. Because of this, healthcare providers may struggle to trust the predictions made by these models, which is important for making informed treatment decisions [94]. Besides this, it complicates regulatory approval processes, as demonstrating reliability and safety becomes more difficult [95]. However, explainable AI (XAI) tools like SHAP (SHapley Additive exPlanations) provide insights into the working mechanism of these models and can show the impact of each variable on model predictions [96]. Overfitting is a limitation of ML models, where they perform well on training datasets, but poorly on external validation. This is particularly concerning with real-world data, where the condition may differ from the training dataset [97]. Furthermore, the development of ML models is resource-intensive and relies heavily on the quality and quantity of data, requiring specialized expertise and substantial computational power to manage [53]. A detailed comparison of ML models and classical statistical models is presented in Table 2.

**Table 2:** Comparison of Machine Learning Methods and Classical Statistical Models.

Aspects	Machine Learning models	Classical Statistical Model
Complexity Handling	Excels at managing complex, non-linear interactions	Suitable for simpler, linear relationships
Interpretability	Low; often considered a "black box"	High; transparent and easier to explain
Adaptability	High; easily retrained and fine-tuned	Low; requires complete re-evaluation for updates
Data Requirements	Requires large and diverse datasets	Effective with smaller datasets
Performance Metrics	Varies significantly; excels in high-dimensional data	Consistent but may underperform in complexity
Feature Engineering	Automated or extensive preprocessing required	Manual feature selection often sufficient
Scalability	Highly scalable for large datasets and distributed systems	Limited scalability for very large datasets
Error Handling	Advanced algorithms handle noise and missing data well	Sensitive to data quality issues
Time Efficiency	Training can be time-consuming for large datasets	Faster for smaller datasets and simpler models
Flexibility	Versatile; supports tasks like classification, regression, clustering	Limited to specific techniques
Domain Knowledge	Minimal domain knowledge required	Relies heavily on domain-specific insights

### 3.8 Selective Case Studies of Successful Predictive Models

Real-world case studies demonstrate specific applications of AI/ML in predicting AMR, showcasing their value through targeted use cases and exemplary practice. These applications target clinical, surveillance, and public health initiatives, demonstrating various successes in analytics. A study utilized pan-genome-based feature selection from over 2000 strains across four species, which enhanced predictive power and enabled analysis of a broader gene set beyond those directly linked to AMR. The implementation of the XGBoost feature selection method significantly increased model accuracy, with approximately 50 % of the selected genes having no known function, suggesting the potential to expand the repertoire of AMR-related genes [98]. DL models, deep ARG for short reads sequences (DeepARG-SS) and Deep ARG for Long read sequences (DeepARG-LS) were developed to enhance the prediction of ARGs from both short-read and full gene sequences. This research combined metagenomic data with a dissimilarity matrix of established ARG categories, resulting in models that achieved superior precision (>0.97) and recall (>0.90). Supported by the comprehensive DeepARG-DB database, these models outperformed conventional sequence-based approaches, effectively reducing false negatives, and enhancing the detection of resistance genes in diverse environmental samples [99].

An ML-based predictive model was developed using WGS data and susceptibility profiles from 1694 *E. coli*, 658 *S. enterica*, 1236 *S. aureus*, and 3528 MTB isolates. The features of PointFinder and ResFinder programs used to predict binary (susceptible/resistant) AMR profiles were utilized to train the models. The study concluded that species-independent models can effectively predict multi-AMR profiles across multiple species without compromising their robustness with the AUC values ranging from 0.90 to 0.95 [100]. Additionally, a mathematical random process model was introduced to investigate the development of antibiotic resistance in bacteria, influenced by the rate of antibiotic consumption. The model was built by combining data on colistin consumption and resistance data in *A. baumannii* in Valencia, Spain. The study concluded that reducing colistin consumption alone may not be sufficient to prevent the escalation of resistance level [101].

Researchers have developed a rapid phenotypic antibiotic susceptibility test that operates independently of bacterial growth, utilizing nanomotion technology to assess bacterial vibrations. Using a supervise d ML model trained on 2762 nanomotion recordings from positive blood culture of 1180 samples, the model achieved a training accuracy ranging from 90.5 % to 100 %. The method achieved an average accuracy of 97.4 % for susceptibility prediction and 94.3 % for resistance prediction, tested across 223 strains [102]. A Bayesian framework was developed to identify MDR bacteria, using a Gaussian mixture model to detect resistance levels against various classes of antibiotics. This model identified correlations in both quantitative MIC and binary susceptibility. Evaluation of *Salmonella* Heidelberg (SH) data from the NARMS, reveals joint resistance to amoxicillin-clavulanic acid and cephalothin, as well as concurrent resistance to ampicillin and cephalothin [103].

Researchers from Vietnam used EHR data from two hospitals to determine the ability of ML models to predict antibiotic resistance among ICU patients. XGBoost, LightGBM, and RF were identified as the top-performing models achieving accuracy levels ranging from 0.890 to 1.000 across both datasets [104]. A study utilized the LR model with backward stepwise predictor selection to predict the risk of MDR infection in cirrhosis patients. The variables included sex, infection type and site, prior use of antibiotics and vasopressors, acute-on-chronic liver failure, use of MELD-Na, and interaction terms. This model achieved an AUROC of 0.68 and has the potential to improve the empirical antibiotic selection [105].

ML-based AMR predictive models against *E. coli* in limited healthcare resource settings, specifically in low and middle-income countries, were developed. WGS and resistance data against ampicillin, ciprofloxacin, and cefotaxime were used for model construction. Validation with data from Uganda, Nigeria, and Tanzania demonstrated reasonable performance; the SVM achieved 87 % accuracy for ciprofloxacin, the LightGBM reached 92 % for cefotaxime and LR attained 94 % for ampicillin [67]. Researchers used deep neural pursuit average activation potential (DNP-AAP) to determine the AMR-associated genes and SNP in the dataset of *Neisseria gonorrhoeae* isolates with WGS data. The prediction AUC ranged from 0.949 to 0.994 for different antibiotics [106]. The SVM model achieved an accuracy ranging from 93 % to 99 % for ARG classification in the dataset of selective gram-negative isolates [107]. Lasso regression algorithms were used to predict colistin resistance in the WGS dataset of *E. coli*. The model achieved an AUC of 0.902 on the validation set and 0.921 on the training set [108]. LR algorithms predict AMR in *Enterobacteriaceae* using the WGS data from 78 clinical isolates, achieving an agreement with standard-of-care phenotypic diagnostics of 90.3 % [109]. Two studies predict carbapenem resistance in *P. aeruginosa* using the GBDT algorithm and supervised machine learning classification using an AdaBoost ensemble model, respectively. The AUC for GBDT was 0.95, while the AdaBoost ensemble model achieved an AUC of 0.925 [110], [111]. A CNN model was developed using raw Raman spectra data to predict resistant phenotypes and ARGs from *K. pneumoniae*. The model achieved an AUC of 0.97, demonstrating significantly higher accuracy than SVM and LR models [112]. Linear classifiers, decision trees, and ensemble classifiers were used to predict rifampicin resistance in tuberculosis using WGS data. The interactive tool, StrUctural Susceptibility PrEdiCTion for RIFampicin (SUSPECT-RIF), achieved a sensitivity of 92.2 %, and specificity of 83.6 %. It is freely available at; https://biosig.unimelb.edu.au/suspect_rif/, (accessed on 25th December 2024) [113]. Ten different ML algorithms were used to predict resistance to ceftazidime, ciprofloxacin, and meropenem based on *P. aeruginosa* gene expression data. For ciprofloxacin, the RF classifier performed well with an F1 score of 0.6, while for ceftazidime and meropenem, the ridge classifier and KNN classifier achieved moderate F1 scores of 0.652 and 0.629, respectively [114].

An LR model was developed to predict the MIC of ciprofloxacin using the acquired ARGs and genome-wide mutational data from *E. coli* isolates, achieving an AUC of 1.0 [115]. This model stands out as one of the most efficient for predicting ciprofloxacin resistant in *E. coli*, surpassing previous studies with reported AUCs of 0.98 [116], and 0.9652–0.9786 [109]. A MBC-Attention (multi-branch convolution neural network architecture and attention) model was utilized to predict the MIC of peptides against *E. coli*. The model achieved a root mean squared error (RMSE) of 0.533 (log µM) and a Pearson correlation coefficient (PCC) of 0.775. The authors concluded that the PCC and RMSE showed 12 % and 13 % improvement, respectively, compared to 17 traditional ML models [117]. An XgBoost model was trained using amino acid k-mer features extraction for AMR prediction in *K. pneumoniae, C. jejuni*, *N. gonorrhoeae,* and *S. enterica*. The author concluded that the amino acid k-mer features extraction-based XgBoost model is more efficient than other feature extraction methods like SNP calling or nucleotide k-mers. Moreover, comparative analysis with other RM showed that the accuracy of the XgBoost model used in the current study (0.95–0,97) was significantly higher than that of models like RR, LR, SVR, and AdaBoost [118].

RF, SVM, and XgBoost models were used for MIC prediction in *A. baumannii* based on k-mer features. The comparative analysis of these ML models showed that the RF model outperformed the other two algorithms, with average AUC values ≥ 0.945 for the 13 antimicrobial agents [40]. Researchers trained five multi-label classification (MLC) methods, including ensemble of classifier chains (ECC), classifier chain (CC), binary relevance (BR), label powerset (LP), and random label space partitioning with label powerset (RD), for predicting MDR in *E. coli*. The ECC approach outperformed the other four methods based on the Jaccard score, precision, recall, and f-score performance matrix [119]. A study proposed an invocational methodology for MIC prediction from WGS data of *Salmonella* species. They trained RF with multilayer perceptron (MLP) and DeepLift, designing a model called “Genome Feature Extractor Pipeline”. This methodology provides a more detailed analysis, achieving an accuracy of > 96 % for MIC prediction [120]. MRSA is a major health concern worldwide. Prompt identification of MRSA helps healthcare providers efficiently control its dissemination in hospital settings. Researchers used an AMRQuest ML-based approach to MALDI-TOF peaks for the presumptive identification of MRSA. This system showed an accuracy of 87.6 %, a specificity of 83.3 %, and a sensitivity of 91.8 % in identifying MRSA [121]. Similarly, RF, SVM, and XgBoost were trained for the identification of MRSA based on k-mer extraction from WGS data. The study revealed that a single two-fold dilution resulted in categorical agreement and essential agreement of > 90 % and 85 %, respectively [122].

### 3.9 AI/ML in Drug Discovery and Design

**Fig. 2:**
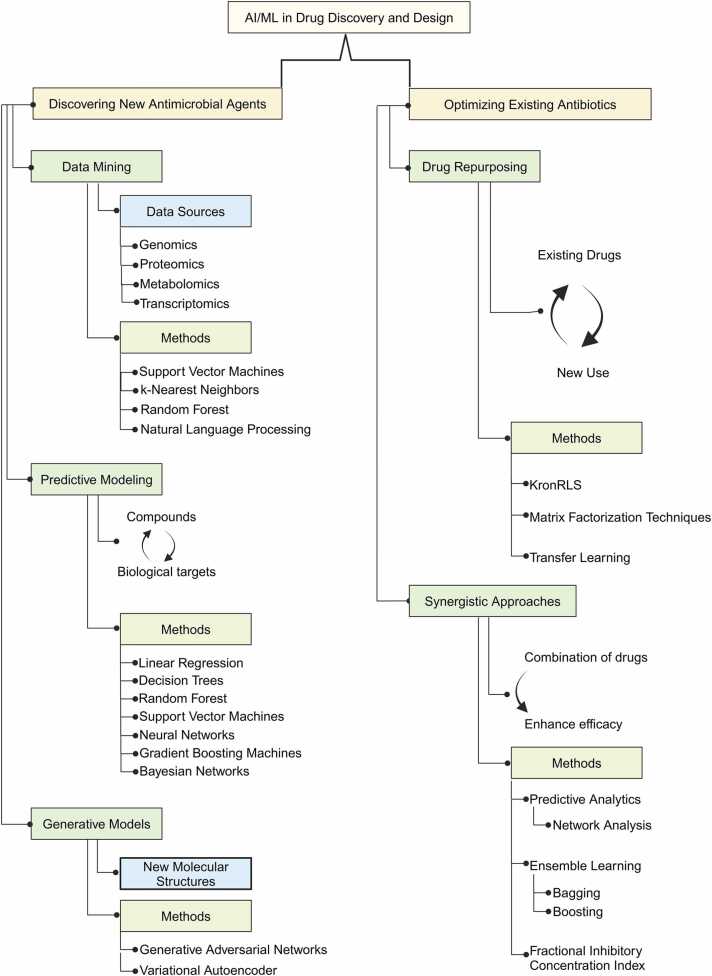
Methods and Applications of Artificial Intelligence and Machine Learning in Drug Discovery and Design.

Advancements in drug discovery and design have been significantly improved by the use of AI and ML (Fig. 2). These technologies have facilitated the identification of new therapeutic targets and novel druggable mechanisms, resulting in more effective drugs with fewer adverse effects, and improving the accuracy of predicting drug safety and efficacy [123]. The AlphaFold model developed by DeepMind, a subsidiary of Google AI, is an AI/ML framework that predicts the 3-D structure of proteins, enhancing the accuracy of drug development [124]. Similarly, pharmaceutical companies utilize IBM's Watson Health platform to accelerate drug design and development. Watson Health analyzes large biological databases to identify new treatments, discover drug targets, and predict the safety and effectiveness of medications [125]. Benevolent AI, a biotech company, is trained on over 2 billion biomedical articles and various data sources to identify new drug targets for rare diseases [126].

In antimicrobial discovery, AI and ML have significantly advanced genome and metabolome mining. Traditional tools like antiSMASH and PRISM are effective in identifying biosynthetic gene clusters (BGCs) but often face challenges with uncharacterized or novel pathways [127], [128]. In contrast, ML-driven tools such as DeepBGC and ClusterFinder utilize sequence-based features to discover new BGC classes, including previously unidentified ribosomally synthesized and post-translationally modified peptides (RiPPs) like deepflavo and deepginsen, by linking distant precursor peptides with biosynthetic enzymes [129].

AI also addresses challenges in metabolomics by enhancing structural inference from mass spectrometry (MS) data, such as molecular formula annotation and retention time prediction [130], [131]. DL algorithms predict biosynthetic routes from chemical structures, integrating genomic and metabolomic data to enable synergistic metabolite structure predictions [132]. Structural elucidation is further enhanced by tools like SMART 2.0, which recently aided in the discovery of symplocolide A, a novel macrolide [133].

Moreover, AI-driven bioactivity prediction models have been developed to identify potential targets, biological activities, and toxicity profiles of natural products. Methods ranging from molecular docking to cheminformatics-based approaches are now enhanced by deep learning models like chemprop, which can predict antibacterial activity [134]. Additionally, natural language processing (NLP) techniques, such as word2vec, encode sequence data into contextual embeddings, enabling the prediction of BGC activities with greater precision [129], [135]. By streamlining discovery, improving structural characterization, and predicting therapeutic potential, AI and ML technologies provide powerful tools for combating AMR and accelerating the development of next-generation therapeutics.

### 3.10 Using AI/ML to Discover New Antimicrobial Agents

Finding new antimicrobial agents is vital for combating AMR. The collaboration between AI/ML technologies and advancements in chemo- and bioinformatics has significantly accelerated this process [136]. AI-based virtual screening is a computer-assisted approach that searches through a large collection of chemicals. Using chemical structure, ML models can predict the antifungal, antiviral, antiparasitic, or antibacterial properties of a compound. This method can save both resources and time by identifying the compounds that require experimental validation [137].

AI algorithms play a vital role in crafting new compounds and optimizing molecular structures to improve antibacterial effectiveness while reducing side effects. This expedites the discovery of novel molecules against resistant strains and provides revolutionary approaches to combat AMR [126]. In addition, AI and ML allow access to valid insights from large data sets of natural products. This approach harnesses Earth's biodiversity to identify potential pharmaceutical candidates that conventional methodologies might otherwise overlook [138].

In the context of predicting novel antimicrobial compounds, researchers trained eight different algorithms including extreme gradient boosting, random forest, deeper neural networks, and gradient boosting classifier, on a dataset of 312 antibiotics versus 936 non-antibiotic drugs. The top four classifiers achieved over 80 % accuracy in both test and blind data using five-fold cross-validation. A soft-voting ensemble was then developed, culminating in the final AdaBoost model, which is now available as an online tool named ABDpred, (http://clinicalmedicinessd.com.in/abdpred/, accessed on 22 July 2024) [139]. In addition, researches developed an ML-based model called AMPSphere that predicts global microbiome-derived antimicrobial peptides (AMPs) by examining 63,410 metagenomes and 87,920 prokaryotic genomes from different habitats. The AMPSphere catalog contains 863,498 predominantly novel peptides, offering valuable insight into peptide evolution and AMP production across various habitats. One hundred AMPs were synthesized to validate the predictions against resistant pathogens and human gut commensals. Of these, 79 were active in the assay, with 63 showing disruption of bacterial cell membranes. This method provides nearly 1 million AMP sequences that might help in the discovery of novel antibiotics [140].

Using experimental data, an AI-based graph neural network facilitated the discovery of chemical substructures responsible for selective antibiotic activity from over 12 million compounds. This strategy led to the discovery of a novel class of antibiotics, demonstrating *in vitro* and *in vivo* antibacterial activity against gram-positive pathogens, including *S. aureus* [141]. Researchers utilized a Wasserstein generative adversarial network with gradient penalty to identify novel AMP candidates from existing AMPs. Through in silico analysis, researchers identified eight candidates (GAN-pep 1–8), which were synthesized and tested. Seven of these candidates showed antibacterial activity by disc diffusion and MIC assays. Among them, GAN-pep-3 and GAN-pep-8 were identified as broad-spectrum antibacterials, particularly effective against carbapenem-resistant *P. aeruginosa* and MRSA. GAN-pep-3, stands out as the most promising candidate, showing very low MICs against all tested bacterial isolates [142]. The list of antimicrobial agents and AMPs, along with their mechanism of action and MIC50/IC50, is detailed in Table 3.

**Table 3:** Example of antimicrobial agents and peptides discovered or designed using artificial intelligence (AI) and machine learning (ML).

Antimicrobial Agent	Methods Used	Target Pathogens	MIC/IC50	Mechanism of Action	Key Properties	References
SPR206	Structure-activity relationship (SAR) models	Carbapenem-resistant *A. baumannii*, *P. aeruginosa, K. pneumoniae*	0.125 mg/L (MIC50)	Targets bacterial membrane	Reduced nephrotoxicity, high efficacy	[143]
QPX9003	AI-guided structure optimization	Carbapenem-resistant *A. baumannii*, *P. aeruginosa, and K. pneumoniae*	0.06 – 0.25 mg/L (MIC50)	Disrupts cell membrane	Improved safety profile, effective at lower doses	[144]
MRX−8	Predictive modeling	MDR *E. coli*, *P. aeruginosa*, *K. pneumoniae*	0.12 – 0.5 mg/L (MIC50)	Disrupts membrane integrity	Lower toxicity, enhanced pharmacokinetics	[145]
Murepavadin	Iterative peptidomimetic design	*P. aeruginosa*	0.12 mg/L (MIC50)	Inhibits lipopolysaccharide transport	High specificity, effective in biofilms	[146]
IB−367 (iseganan)	AI peptide design	Oral infections (Gram-positive/Gram-negative pathogens)	0.13–64 µg/ML (MIC against gram-positive) 0.06–8 µg/ML (MIC against gram-negative)	Membrane disruption	Safe but limited efficacy in clinical settings	[147]
SCH−79797	ML-based dual mechanism profiling	Gram-positive/Gram-negative	1–6 µg/ML (MIC)	Folate metabolism and membrane integrity	Broad-spectrum efficacy	[147], [148]
Bactenecin	Machine-learning classifer	*E. coli,P. aeruginosa, S. typhimurium, S. aureus, L. monocytogene, C. xerosis, S. pyogene*	8 64 μg/ML (MIC)	Membrane disruption	Broad-spectrum efficacy	[149]
DP7	AI-driven sequence optimization	*S. aureus*	16 mg/L (MIC)	Disrupts biofilms and enhances immunity	Low cytotoxicity, strong biofilm activity	[150]
Lead AMPs	PanCleave random forest modeling	*E. coli, K. pneumoniae, A. baumannii, P. aeruginosa, S. aureus,*	8–64 μmol/L (MIC)	Membrane permeabilization	Low toxicity, high specificity	[151]
HydrAMP-designed AMPs	Conditional variational autoencoder	MDR Gram-positive and Gram-negative pathogens	4 – 512 μg/ML (MIC)	Membrane disruption	Potent and structurally diverse	[152]
Yoshida et al. generated 44 AMPs	Genetic algorithm-based machine learning	*E. coli*	< 4.1 μM (IC50)	Membrane disruption	Low toxicity	[153]
Cao et al. designed (A−222) AMPs	Deep generative model	*E. coli*, *P. aeruginosa, S. aureus*, *B. subtilis*, *S. maltophilia*, *B. thuringiensis*, *L. enzymogenes*	16–256 μg/ML (MIC)	Membrane disruption	Broad-spectrum efficacy	[154]
Nagarajan et al. designed 10 AMPs	Long short-term memory (LSTM) language model	*E. coli, A. baumannii, K. pneumoniae, P. aeruginosa, S. aureus,*	≤ 128 μg/ML (MIC)	Membrane disruption	Effective against Carbapenem-resistant, Extended-spectrum β-lactamase, and Methicillin-resistant strains	[155]
Huang et al. designed 54 AMPs	Deep learning algorithms	*E. coli, A. baumannii, P. aeruginosa, S. aureus, S. haemolyticus*	≤ 200 μg/ML (MIC)	Membrane disruption	Effective against MDR pathogens	[156]
Porto et al. designed 8 AMPs	Genetic algorithm	*E. coli, A. baumannii, P. aeruginosa, S. aureus, K. pneumoniae, S. pyogenes, L. ivanovii, E. faecalis* and *yeast*	6.25–100 μg/ML (MIC)	Membrane disruption and hyperpolarization	Both bactericidal and antifungal activity	[157]
Ma et al. designed 181 AMPs	Neural network models	*E. coli, A. baumannii, P. aeruginosa, S. aureus, K. pneumoniae, E. faecalisc, S. epidermidis*	2–200 μM (MIC)	Membrane disruption	Broad-spectrum efficacy	[158]
Dean et al. designed 6 AMPs	Variational autoencoder.	*E. coli, A. baumannii, S. aureus*	< 70 μg/ML (IC50)	Membrane disruption	dose-responsive antimicrobial activity	[159]
Dean et al. designed 38 AMPs	Variational autoencoder.	*E. coli, P. aeruginosa, S. aureus*	0.5–128 μM (MIC)	-	-	[160]
Tucs et al. designed 5 AMPs	Generative adversarial network	*E. coli*	3.1–50 μg/ML (MIC)	Membrane disruption	Twice as strong as ampicillin	[161]
Capecchi et al. designed 8 AMPs	Neural language model	*E. coli, A. baumannii, S. aureus, P. aeruginosa, S. maltophilia, E. cloacae, B. cenocepacia, S. epidermidis*	≤ 64 μg/ML (MIC)	Membrane disruption	Effective against MDR strains	[162]

### 3.11 Optimizing existing antibiotics for combating resistant strains

Drug repurposing and combination therapies are two of the most important approaches for optimizing existing antibiotics. AI/ML technologies have the potential to accelerate the process of drug repurposing by efficiently identifying existing remedies with antibacterial properties. These technologies facilitate the analysis of chemical and biological characteristics of these drugs, enabling the targeting of resistant pathogens and broadening the therapeutic applications of established medications [163].

Researchers at the Massachusetts Institute of Technology (MIT) employed a DL model to discover halicin as a potential antibiotic, originally developed as a diabetes drug. The AI model was trained on approximately 2335 molecules, including Food and Drug Administration (FDA)-approved drugs and natural products. The testing was conducted using the Drug Repurposing Hub, which contains around 4498 compounds. Halicin demonstrated potent antibacterial activity, especially against drug-resistant bacteria like *C. difficile*, *A. baumannii*, *M. tuberculosis*, and carbapenem-resistant *Enterobacteriaceae*. It disrupts the electrochemical gradient inside bacterial membranes, thereby decreasing the likelihood of resistant development [164]. Another significant example is Abaucin, a compound initially developed for diabetes treatment. However, during the screening of Drug Repurposing Hub, Abucin was found to be an effective medication against pan-resistant *A. baumannii* [165].

AI/ML technologies limit the rise of resistance and improve antibiotic efficacy by rapidly screening antibiotic synergism against resistant strains [166]. An AI/ML-based platform known as IDentif.AI focuses on combating AMR to optimize combination therapies against infectious diseases [166]. Researchers tested 155 drug combinations, including 12 FDA-approved compounds, at various concentrations against carbapenem-resistant *Enterobacteriaceae* (CRE). The sub-lethal dose of bleomycin (an anticancer drug) in combination with meropenem showed synergistic bactericidal activity against CRE and was safe for mammalian cells. A similar effect of bleomycin in combination with ertapenem and imipenem was observed, suggesting that AI-guided combination therapy is a promising approach for combating AMR [167].

## 4 Clinical Decision Support Systems (CDSS)

Clinical Decision Support Systems (CDSS) are important tools in modern healthcare settings. They utilize patient data and computer-based knowledge to assist physicians in making more informed decisions. The tools used in these systems include pre-defined treatment plans, medication alerts, reminders for clinical tasks, and online patient portals. They improve healthcare practices by offering treatment and diagnostic strategies based on the optimized protocols [168].

The prescription of antibiotics based on the internationally accepted standardized protocol helps reduce the emergence of resistant strains. AI/ML-based CDSS guides physicians in prescribing the appropriate antibiotics [169]. AI tools assist in identifying causative agents, profiling susceptibilities, and providing recommendations based on the regional AMR scenario [170]. Their main advantage is that it suggests narrow-spectrum antibiotics, minimizing the overuse and misuse of antibiotics, and thereby preventing the emergence of resistant pathogens [171], [172]. It plays an important role in improving antimicrobial stewardship by detecting inadequate treatments and endorsing the appropriate use of antibiotics. For example, if a viral infection instead of a bacterial were detected, it alarms for discontinuation of antibiotics and suggests the appropriate anti-viral therapy [173].

**Fig. 3:**
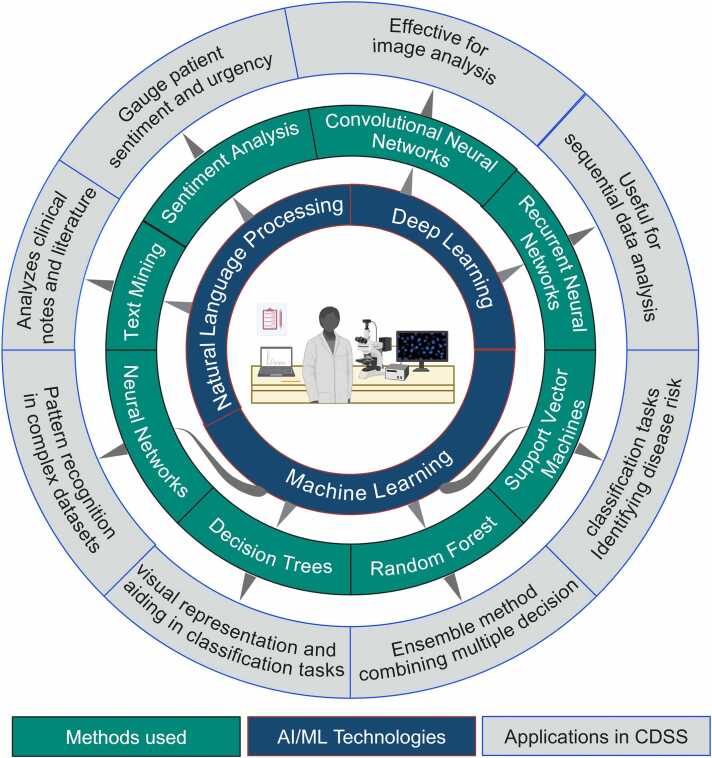
Artificial Intelligence and Machine Learning Technologies, Methods, and Applications in Clinical Decision Support Systems.

AI/ML-based CDSS contains tools for tracking outcomes and feedback, and they can update recommendations when better outcomes are found [174]. The Nivel, Pacmed, and Leiden University Medical Center (LUMC) established ML-based CDSS for medical doctors to evaluate antibiotic prescriptions, using urinary tract infections as cases. They used decision tree classifiers to predict treatment outcomes, which helped to improve the success rate of treatment from 75 % to 85 % in a prospective study (P < 0.001). It suggests that CDSS based on AI/ML tools can improve healthcare quality (Fig. 3) [175].

## 5 Surveillance and epidemiology

Surveillance and epidemiology are closely related fields that study health patterns in populations over time. Surveillance involves regularly collecting, analyzing, and interpreting health data, which is essential for planning, operating, and assessing public health strategies [176]. Epidemiology focuses on diseases and their causative agents, aiming to find pathogens and reduce risk factors. These fields are important for outbreak management, optimizing resource utilization, and guiding targeting prevention efforts [176]. Organizations like the Centers for Disease Control and Prevention (CDC) and WHO, with the expertise of epidemiologists, effectively used these strategies in response to outbreaks such as COVID-19 [177].

Epidemiologists use AI/ML tools to identify resistant strains from various sources, including hospitals, animal husbandries, and the environment (Table 4) [178]. For instance, AI and ML methods can analyze a large amount of clinical data, including EHR, laboratory reports, and genetic information, to rapidly detect emerging resistance trends [179]. ML-based Predictive models can systematically analyze archived AST data, genomic information, and antibiotic consumption usage patterns to forecast future AMR outbreaks [180]. Several AI/ML-powered AMR surveillance tools are actively utilized worldwide. For example, the CDC-supervised NARMS at present applies AI/ML techniques to evaluate data about AMR in humans and animals and retail meat products from the United States, yielding significant insights into emerging threats [181]. Similarly, the GLASS collects global AMR data to support public health strategies with evidence-based information [3].

**Table 4:** AI/ML-Based Surveillance and Epidemiology of Antimicrobial Resistance (AMR).

AI/ML Technologies	Application	Use Cases	Example Tools/Frameworks	Current Trends	Advantages	Disadvantages
Machine Learning (ML)	Analyze large datasets to identify trends.	Predicting AMR trends based on historical antibiotic usage.	Scikit-learn, TensorFlow	Growing use of automated ML tools.	Effective in recognizing patterns in complex data.	Requires large datasets for training.
Random Forest (RF)	Identify factors associated with AMR.	Analyzing correlations between antibiotic usage and resistance emergence.	R, Python (Scikit-learn)	Increasing integration with genomic data.	High accuracy, reduces overfitting.	Can be slow and ineffective for real-time predictions.
Support Vector Machines (SVM)	Classify resistance based on genomic data.	Classifying pathogens based on resistance patterns in genomic data.	R, Python (Scikit-learn)	Application in real-time surveillance.	Effective for high-dimensional data.	Sensitive to noise and outliers.
Ensemble Methods	Improve prediction accuracy.	Using multiple models to enhance prediction of AMR.	XGBoost, LightGBM	Enhanced focus on combining methods for robustness.	Combines multiple models for better performance.	More complex to implement and interpret.
Bayesian Networks	Model relationships and uncertainty in data.	Understanding the relationships between different resistance mechanisms.	PyMC3, bnlearn	Increasing use in probabilistic modeling.	Good for probabilistic reasoning.	Can be complex to build and require expert knowledge.
Clustering Algorithms	Identify resistance patterns among pathogens.	Classifying pathogens into groups based on resistance profiles.	R (factoextra), Python (Scikit-learn)	Use in outbreak detection for pattern recognition.	Useful for exploratory data analysis.	May require tuning of parameters
Deep Learning (DL)	Recognize complex patterns in data.	Analyzing genomic sequences for resistance genes.	TensorFlow, Keras	Increasing use in image analysis for diagnostics.	Excels with large datasets.	Requires substantial computational resources.
Convolutional Neural Networks (CNNs)	Analyze medical images.	Detecting anomalies in pathogen cultures or patient imaging.	TensorFlow, PyTorch	Use in analyzing diagnostic imaging data.	Highly effective for image-related tasks.	Overfitting on small datasets.
Recurrent Neural Networks (RNNs)	Analyze sequential data (e.g., patient data).	Monitoring patient vitals and their relation to AMR development over time.	TensorFlow, Keras	Application in continuous monitoring systems.	Effective for time-series analysis.	Can be difficult to train effectively.
Natural Language Processing (NLP)	Analyze unstructured clinical data.	Evaluating clinical notes to identify resistance patterns.	NLTK, SpaCy, Hugging Face Transformers	Growing application in automated clinical documentation.	Extracts insights from text efficiently.	Challenges with context and nuance in language.
Text Mining	Extract insights from literature and records.	Identifying new resistance genes from published research articles.	R (tm), Python (NLTK)	Increasing focus on automated literature reviews.	Useful for identifying emerging trends.	Requires comprehensive natural language understanding.
Sentiment Analysis	Gauge urgency and perception of AMR threats.	Evaluating public health communications regarding AMR threats.	R (sentimentr), Python (TextBlob)	Enhanced monitoring of public health responses.	Provides insights into public and clinician attitudes.	Interpretation can be subjective.
Federated Learning	Collaborative model training without data sharing.	Collaborating on AMR data across institutions while maintaining privacy.	TensorFlow Federated	Increasing focus on privacy-preserving techniques.	Protects sensitive health data.	More complex coordination required.
Transfer Learning	Leverage knowledge from related domains.	Adapting models trained on general healthcare data for AMR-specific tasks.	PyTorch, TensorFlow	Growing interest in cross-domain applications.	Reduces need for labeled data in AMR contexts.	Requires suitable pre-trained models.
Graph-Based Learning	Model relationships between pathogens.	Analyzing networks of resistance genes and their interactions.	DGL, PyTorch Geometric	Use in understanding pathogen interactions.	Effective for representing complex interactions.	Requires graph construction knowledge.
Time-Series Analysis	Forecast trends based on historical data.	Forecasting resistance trends based on antibiotic usage over time.	Statsmodels, Prophet	Increasing application in predictive analytics.	Effective for identifying temporal patterns.	May require careful preprocessing.
Generative Adversarial Networks (GANs)	Generate synthetic data for model training.	Enhancing datasets for training AMR predictive models.	TensorFlow, PyTorch	Emerging use in synthetic data generation.	Useful for augmenting small datasets.	Complex to train effectively.

In addition, AI/ML tools can identify intricate patterns in a wide range of data thereby providing insight into the mechanisms driving emerging drug resistance [172]. PathogenWatch, an AI-based tool, enables real-time analysis and visualization of microbial genome sequences in both phylogenetic and geographical contexts, improving the tracking and monitoring of pathogen outbreaks [182]. AI/ML-powered real-time monitoring informs the timely adaptation of treatment and containment strategies. For instance, the researchers developed ScanGrow, a software designed to automate the capture and analysis of bacterial broth images in microplates by training a DL model. It is a cost-effective alternative to conventional spectrophotometric plate readers and enables efficient monitoring of bacterial growth and evaluation of antibiotic effects [183].

## 6 Challenges and future directions

AI/ML holds great potential to profoundly transform healthcare, especially AMR surveillance, epidemiology, and outbreak detection and response. However, several limitations remain, such as ethical considerations related to the use of genetic and clinical data, highlighting the importance of appropriate data collection and adherence to informed consent protocol. Privacy and security concerns regarding patient data are critical to ensure that AI/ML systems comply with regulations such as the Health Insurance Portability and Accountability Act (HIPAA) in the U.S., which safeguards sensitive health information. Model explainability and accountability are also among the main challenges for both governments and individual institutions. Furthermore, biases and heterogeneity of EHR data from various sources limit the effectiveness of AI/ML tools in mitigating AMR.

Future directions in the field of AMR combating should focus on refining the current models for greater accuracy and scalability. Special attention to the analysis of real-time data from clinical, environmental, and genomic datasets is required for more accurate predictions of AMR outbreaks. To ensure trust in insight provided by AI, XAI models need to be developed. AI-based AST diagnostic technologies should be developed for the rapid identification of resistant strains. Collaboration efforts between bio-informaticians, microbiologists, and clinicians are required for developing personalized treatment strategies, based on patients' profiles and specific pathogens.

## 7 Conclusions

This review explores the potential transformative impact of AI/ML on AMR management, encompassing areas such as enhanced surveillance, predictive modeling, and outbreak response. These technologies empower healthcare professionals and policymakers to monitor resistance trends and identify complex patterns in AMR dynamics by utilizing large-scale data from diverse sources. AI/ML-powered early warning systems and predictive analytics significantly enhance our ability to respond promptly to drug-resistant outbreaks. However, addressing key challenges such as ethics, privacy, and bias is essential in this field. Joint initiatives such as tech developers, healthcare operators, and regulatory authorities are required to overcome these challenges and leverage AI/ML-based tools to combat AMR.

## CRediT authorship contribution statement

**Xiaohui Li:** Writing – review & editing. **Qiao-Li Lv:** Writing – review & editing, Supervision, Conceptualization. **Rahat Ullah Khan:** Writing – review & editing. **Mujeeb Ur Rahman:** Writing – review & editing. **Bin Xu:** Writing – review & editing, Supervision, Conceptualization. **Hazrat Bilal:** Writing – original draft, Visualization, Formal analysis, Conceptualization. **Muhammad Shafiq:** Writing – review & editing. **Wenjie Fang:** Writing – review & editing. **Muhammad Nadeem Khan:** Writing – original draft, Visualization, Conceptualization. **Sabir Khan:** Data curation.

## Declaration of Competing Interest

The authors declare that they have no known competing financial interests or personal relationships that could have appeared to influence the work reported in this paper.
